# Dynamics of abdominal symptoms during the start of a new therapy with elexacaftor/tezacaftor/ivacaftor using the novel CFAbd-day2day questionnaire

**DOI:** 10.3389/fphar.2023.1167407

**Published:** 2023-10-25

**Authors:** Jochen G. Mainz, Anton Barucha, Pu Huang, Lilith Bechinger, Franziska Duckstein, Louise Polte, Pauline Sadrieh, Lutz Nährlich, Olaf Eickmeier, Suzanne Van Dullemen, Patience Eschenhagen, Carsten Schwarz, Stefan Lüth, Carlos Zagoya, Ute Graepler-Mainka

**Affiliations:** ^1^ CF-Center, Klinikum Westbrandenburg, Brandenburg an der Havel, Germany; ^2^ Medizinische Hochschule Brandenburg (MHB), Universitätsklinikum, Brandenburg an der Havel, Germany; ^3^ CF Center for Children Justus-Liebig-Universität Gießen, Universitätsklinikum Gießen-Marburg GmbH, Gießen, Germany; ^4^ Division of Allergy, Pulmonology and Cystic Fibrosis, Department for Children and Adolescents, University Hospital, Frankfurt, Germany; ^5^ CF-Center, Klinikum Westbrandenburg, Potsdam, Germany; ^6^ Department of Gastroenterology, Brandenburg Medical School (Theodor Fontane), Brandenburg an der Havel, Germany; ^7^ Children’s Hospital, Eberhard-Karls-University, Tübingen, Germany

**Keywords:** gastrointestinal, patient-reported outcome measure, prospective, symptom score, diary, CFAbd-Score

## Abstract

**Background:** Elexacaftor–tezacaftor–ivacaftor (ETI) is a novel, highly effective CFTR modulator combination proven to enhance lung function and body weight in people with cystic fibrosis (pwCF) carrying a F508del mutation. Recently, we revealed significant reductions in abdominal symptoms (AS) in German, British, and Irish pwCF after 24–26 weeks of ETI using the CFAbd-Score, the first patient-reported outcome measure (PROM) specifically developed and validated for pwCF following FDA guidelines. Notably, many pwCF reported marked changes in their AS during the first days of the new treatment. To capture these immediate effects, we developed the CFAbd-day2day, a CF-specific GI-diary, following FDA and COSMIN guidelines.

**Aim:** To prospectively capture the immediate dynamics of AS using the CFAbd-day2day 14 days before and 14–28 days after ETI initiation. In addition, we aim to provide validation steps of the novel PROM concerning sensitivity to changes.

**Methods:** To develop the CFAbd-day2day, focus groups (community voice = pwCF and their proxies and CF specialists from different fields) were repeatedly consulted. Before and during the new ETI therapy, pwCF prospectively scored AS on a daily basis with the CFAbd-day2day.

**Results:** Altogether, 45 pwCF attended in five CF centers prospectively completed the CFAbd-day2day before (mean ± sd:14 ± 7 days) and after (mean ± sd: 28 ± 23 days) ETI initiation. On the one hand, cumulative scores significantly decreased during the 3–4-week time frame after ETI initiation, compared to 2 weeks prior to therapy. On the other hand, many patients who revealed a relatively stable level of AS before ETI reported changes during the first days of treatment with the highly effective CFTR modulators. Factors like pain and flatulence increased in up to 21% of patients during the first 14 days of therapy, but they improved during days 15–27.

**Conclusion:** The CFAbd-day2day, specifically developed and in the process of validation to prospectively capture GI symptoms in pwCF, provides new substantial insights into the dynamics of AS in pwCF receiving a new treatment with ETI. This novel tool is also helpful in prospectively monitoring patients with specific GI problems. International implementation and further validation steps of the diary are ongoing.

## 1 Introduction

For long, abdominal involvement in cystic fibrosis (CF), the most common lethal inherited disease of the Caucasian population, received little attention. Since the availability of pancreatic enzyme supplementation therapy (PERT) in mostly (Ratjen and Doring, 2003) pancreatic insufficient patients, pulmonary infection and lung destruction became the major reason for premature death in approximately 90% of people with CF (pwCF) (Martin et al., 2016; Deutsches Mukoviszidose-Register Berichtsband, 2020). The causative gene defect results in abnormal production and function of the cystic fibrosis transmembrane conductance regulator (CFTR) protein, crucially affecting both the respiratory and gastrointestinal systems. Apart from the upper and lower airways, the ATP-gated anion channel is highly expressed in the pancreas, gut, and bile ducts, leading to a CF-specific pattern of gastrointestinal (GI) complaints (Ooi et al., 2016; Freedman et al., 2018). In addition to exocrine pancreatic insufficiency, present from birth in approximately 85% of pwCF, impaired intestinal passage by meconium ileus, distal intestinal obstruction syndrome (DIOS), and constipation are typical complications in pwCF (Munck et al., 2016; Ooi and Durie, 2016; Stefano et al., 2022). These are complemented by factors like intestinal dysbiosis caused by frequent antibiotic treatments, cough-associated reflux, and maldigestion, resulting in malresorption of nutrients, which then allow bacterial fermentation, gases, and diarrhea as principal symptoms of untreated exocrine pancreatic insufficiency (Caley et al., 2023a). Finally, endocrine liberation of insulin is also hampered by the destruction of pancreatic beta cells, leading to CF-related diabetes in a substantial proportion of pwCF during adulthood (Caley et al., 2023b).

Introduction of highly effective CFTR modulator therapies (HEMTs) in pwCF carrying a rare gating mutation like G551D over a decade ago revealed that correction and potentiation of the defective and/or malfunctioning CFTR channel have marked effects on the health status of pwCF beyond pulmonary function (Ramsey et al., 2011; Davies et al., 2013; Bodewes and Wilschanski, 2018; King et al., 2021). Patients substantially gained weight and thrived if therapy was introduced in childhood (Davies et al., 2016). At the same time, from personal experience with our patients (Mainz et al., 2018), we learned that HEMTs had effects on abdominal symptoms (AS). However, the lack of validated CF-specific PROMs focusing on GI involvement impeded former rigorous research to adequately assess the apparently substantial changes during early HEMT with ivacaftor in pwCF carrying a gating mutation.

To fill this gap, we developed the CFAbd-Score in different steps following FDA guidelines for the development of a PROM (U.S. Department of Health and Human Services Food and Drug Adminitration, 2019), including CF patients and their families (community voice), as well as professional CF specialists at several time points. Initially, the PROM had been named JenAbd-Score (Tabori et al., 2017a), and after condensing it from a 5-sided questionnaire to a one-sided PROM, which comprises 28 symptoms grouped in five domains, it was renamed “CFAbd-Score” (Tabori et al., 2017b; Jaudszus et al., 2019; Mainz et al., 2023; Jaudszus et al., 2022; Mainz et al., 2022; Caley et al., 2023b). Presently, it is available in eleven languages and implemented in more than 25 studies around the world (Boon et al., 2020; Mainz et al., 2023; Ng et al., 2021; Mainz et al., 2022; Raun et al., 2022).

Recently, we implemented the CFAbd-Score in an international study with 107 pwCF from Germany and the UK before and at a median of 24 weeks during therapy with elexacaftor–tezacaftor–ivacaftor (ETI). Therein, ETI was found to substantially improve the total CFAbd-Score and its five domains: “pain,” “GERD,” “disorders of bowel movement,” “disorders of appetite,” and “quality of life impairment” (Mainz et al., 2022). Quite a similar improvement pattern was observed during ETI in a parallel study administering the CFAbd-Score to 103 Irish and British pwCF before and after 1, 2, 6, and 12 months of ETI (Mainz et al., 2023). In both studies, the CFAbd-Score was observed to have high sensitivity to the changes induced by the new therapy with ETI, which is considered a game changer in CF. A recent multicenter study in the United States also found a significant reduction of GI symptoms in 263 pwCF receiving ETI. However, unlike results obtained with the CF-specific CFAbd-Score (Mainz et al., 2022; Mainz et al., 2023), changes assessed with questionnaires evaluated and validated for other GI pathologies, but not for pwCF [patient assessment of upper gastrointestinal disorders-symptom (PAC-SYM), patient assessment of constipation-symptom (PAC-SYM), and patient assessment of constipation-quality of life (PAC-QOL)], did not reach the level of clinical significance (Schwarzenberg et al., 2022).

Nevertheless, during the early phases after HEMT introduction, patients reported experiencing many GI symptoms, which may not be adequately represented by a PROM that addresses GI symptoms retrospectively with a 2-week recall period. Furthermore, after HEMT initiation some symptoms could arise more frequently and markedly, but only for quite a short period of time, which may not be adequately recorded with a questionnaire focused on capturing the overall frequency of GI symptoms over the past 14 days.

Consequently, based on the CFAbd-Score, we developed the CFAbd-day2day questionnaire, a prospective diary PROM. This questionnaire is being validated following FDA and COnsensus-based Standards for the selection of health Measurement INstruments (COSMIN) guidelines (Mokkink et al., 2010; U.S. Department of Health and Human Services Food and Drug Adminitration, 2019). Analogous to the development of the CFAbd-Score, pwCF and their families, as well as professional CF specialists (community voice), were included to optimize the wording and structuring of the PROM at several time points. The resulting prospective CF-specific GI-symptom diary CFAbd-day2day^©^ is suitable for closely recording AS.

As the selection and wording of questions included in the CFAbd-Score have been found to be highly sensitive for capturing and quantifying GI symptoms in pwCF receiving a new ETI therapy (Mainz et al., 2023; Mainz et al., 2022), substantial changes concerned rewording of questions to allow prospective GI symptom inquiring on a daily basis. Furthermore, leaving room for individual comments and observations not covered by the questionnaire, such as eating habits and changes in PERT, appeared essential for the CFAbd-day2day.

The aim of this study was to prospectively assess changes in abdominal symptoms on a daily basis during a new, highly effective CFTR-modulating ETI therapy in pwCF. Specifically, we aimed to capture GI symptoms for a period of up to 14 days prior to commencing the new therapy, as well as during 14–28 days of the new therapy, using the novel CFAbd-day2day^©^ questionnaire.

## 2 Methods

### 2.1 Participants

A total of 51 pwCF attending five German CF care centers in Brandenburg an der Havel/Potsdam (n = 22), Tübingen (n = 15), Gießen (n = 7) and Frankfurt am Main (n = 7) were prospectively considered to complete the CFAbd-day2day^©^ before and after ETI initiation. Of note, 10 pwCF recruited at the CF centers in Brandenburg an der Havel/Potsdam for this project, which focuses on prospective short term changes in the CFAbd-day2day, were also included in the study previously published in 2022 (Mainz et al., 2022). Further inclusion criteria were: confirmed evidence of CF by two positive sweat tests and/or detection of two CFTR mutations, carrying at least one allele with F508del, as a requirement for ETI therapy initiation. Exclusion criteria were: inability to comply with the study procedures or assessments and below 6 years of age. Eligible subjects were included independent of their severity of pulmonary function (FEV1pred), airway colonization with specific pathogens, and comorbidities. A history of concomitant GI manifestations as well as food allergy or intolerance was recorded. Data acquisition was performed using *pseudonymization* and after written consent from the parents/legal guardian or from the pwCF themselves if their age was above 18 years.

### 2.2 Assessment of symptoms—CFAbd-day2day^©^

Abdominal symptoms were recorded daily using the CFAbd-day2day^©^ questionnaire. The CFAbd-day2day^©^ is a diary version of the CFAbd-Score. Therefore, the CFAbd-day2day^©^ also comprises 28 items grouped into five domains. However, in addition to the modified recall period, some questions have been adapted to prospectively focus on each observational day. The questionnaire also includes an optional comments section for recording changes in dietary habits, enzyme and medication use, and menstrual symptoms. A major intrinsic advantage of the diary character is the ability to record not only symptom frequencies but also their daily intensity. Printed copies of the questionnaire were issued to the participants through the local CF care providers and/or research coordinators. Scoring and analyses of completed, pseudonymized questionnaires were centrally performed at the CF center in Brandenburg an der Havel, Germany.

### 2.3 Statistical analyses

Individual baseline values for each of the 28 items included in the CFAbd-day2day^©^ questionnaire were obtained by computing medians of each item over the time frame prior to ETI therapy initiation for each pwCF. Dynamics of symptoms were assessed by computing daily proportions of subjects reporting either improvement or worsening with respect to the aforementioned baseline values for each of the 28 symptoms assessed with the CFAbd-day2day^©^ questionnaire. Furthermore, within-subject averaged absolute daily variation measures were computed for each subject and each questionnaire item to quantify the levels of variation over three time frames: prior to ETI therapy and 2 and 4 weeks after ETI therapy initiation. These measures were computed by averaging the absolute changes experienced by each patient between consecutive days in each item over the aforementioned time frames. Additionally, the maximum deviation with respect to the median in each item was identified for each patient within each time frame. In order to find out the period of time (prior to ETI therapy and 2 and 4 weeks after ETI therapy initiation) at which symptoms reported by the cohort underwent maximum changes, exploratory analyses to compare medians of these measures of variability were conducted using non-parametric Wilcoxon signed-rank tests. Effects of ETI therapy on cumulative CFAbd-day2day responses were assessed by mapping the frequency of events registered by each patient for each item in the CFAbd-day2day^©^ with the following scale: not at all → 0, once → 1, 2–3 times → 2, 4–7 times → 3, more than 7 times → 4, and daily → 5. Afterward, items were grouped into five different domains as defined for the 2-week CFAbd-Score (Jaudszus et al., 2019). Scores for each domain and a total score were calculated for both the pre-ETI time frame and the 2-week time period involving the third and fourth weeks after ETI therapy initiation. Comparisons of domains and total scores were conducted using non-parametric Wilcoxon signed-rank tests. Prior to these tests, normality assumptions were tested using quantile–quantile plots as well as the Shapiro–Wilk test.

## 3 Results

A total of 51 pwCF were recruited for this study, of which 50 pwCF completed the questionnaire prior to ETI initiation (median: 15 days, IQR [14, 15] days). 45 (90%) of them completed the questionnaire after ETI initiation (median: 25 days, IQR [25, 27] days). Detailed information about the rates of daily answers is provided in Table 1. All 45 pwCF (median age: 10 [6–55] years) who completed the questionnaire during both time frames (Brandenburg an der Havel/Potsdam (40%), Gießen (16%), Tübingen (31%), and Frankfurt am Main (13%)), were included in the final analysis. In this cohort, 30 (66.7%) pwCF were female and 15 (33.3%) were male (Table 2). The mean time frame of included patients who completed the diary was (mean ± sd) −14 ± 7 days and 28 ± 23 days prior to and after commencing ETI therapy, respectively.

**TABLE 1:** Distribution of responses in regard to relevant time frames.

	Time frame	Proportion of pwCF
Pre-ETI		
	14 days	38/50 (76%)
	10–13 days	4/50 (8%)
	7–9 days	5/50 (10%)
	1–6 days	3/50 (6%)
During ETI		
	27 or more days	29/50 (58%)
	21–26 days	9/50 (18%)
	14–20 days	3/50 (6%)
	10–13 days	3/50 (6%)
	7–9 days	0/50 (0%)
	1–6 days	1/50 (2%)

**TABLE 2:** Demographics and clinical characteristics of pwCF included in this study.

Demographics total (n) age (median, range)	45 10 (6–55) years
Age <18	37 (82.2) n (%)
Age ≥18	8 (17.8)
Female	30 (66.7)
Male	15 (33.3)

Genotype	
F508del homozygous	25 (55.5%)
F508del heterozygous	19 (42.2%)
F508del/unknown	1 (2.2%)

Previous CFTR modulator therapy	
Yes	20 (44.4%)
No	25 (55.5%)
Lumacaftor + ivacaftor	16 (35.5%)
Tezacaftor/ivacaftor + ivacaftor	3 (6.7%)
Ivacaftor	1 (2.2%)

CF-associated diseases/conditions	
Exocrine pancreatic insufficiency	Yes: 42/45 (93.3%)
	No: 3/45 (6.7%)
Meconium ileus	Yes: 8/45 (17.8%)
	No: 37/45 (82.2%)
Distal intestinal obstruction syndrome (DIOS)	Yes: 0 (0%)
	No: 45 (100%)
CF-related diabetes (CFRD)	Yes: 3/45 (6.7%)
	No: 36/45 (80%)
	Unknown: (13.3%)
CF-related liver disease (CFLD)	Yes: 9/45 (20%) Liver fibrosis: 4/45 (8.9%)
	Secondary biliary cirrhosis: 1/45 (2%)
	Hepatic steatosis grade 1: 1/45 (2%)
	Unspecified: 3/45 (6.7%)
	No: 30/45 (66.7%)
	Unknown: 6/45 (13.3%)
Report on food allergies or intolerances	Yes: 8/45 (17.8%)
	No: 37/45 (82.2%)
Lung function (FEV1%pred) (conducted in 41 patients^a^)	88.3% ± 17.1%

Body mass index (BMI)	
Patients ≥18 years of age, n = 8 (17.8%) BMI (mean ± sd)	23.9 ± 4.0 kg/m^2^
Patients <18 years of age, n = 37 (82.2%) BMI-for-age z-scores (mean ± sd)	−0.34 ± 1.0

^a^ Baseline FEV_1_%pred values from four pwCF were not available.

### 3.1 Dynamics of symptoms

**FIGURE 1:**
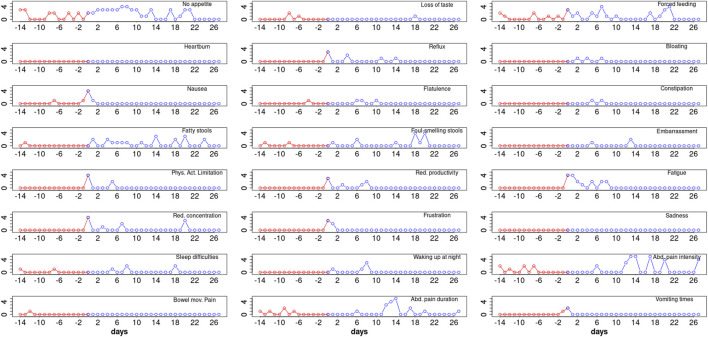
CFAbd-day2day^©^ protocol from a single CF patient (8 years old, male, homozygous for F508del, pancreatic-insufficient, and pre-treated with Orkambi) revealing baseline symptoms prior to ETI (red) as well as evolution of the daily burden of GI-related symptoms during the first 27 days of therapy (blue) for 24 of the 28 items included in the CFAbd-day2day^©^ (*y*-axis represent score responses on a 0–5 Likert scale, where higher scores quote a higher burden of symptoms).

There was a high between- and within-subject variability in the responses of patients, and the dynamics over the considered time periods followed a rather individual profile in all 28 symptoms assessed with the CFAbd-day2day^©^ (Figure 1).

**FIGURE 2:**
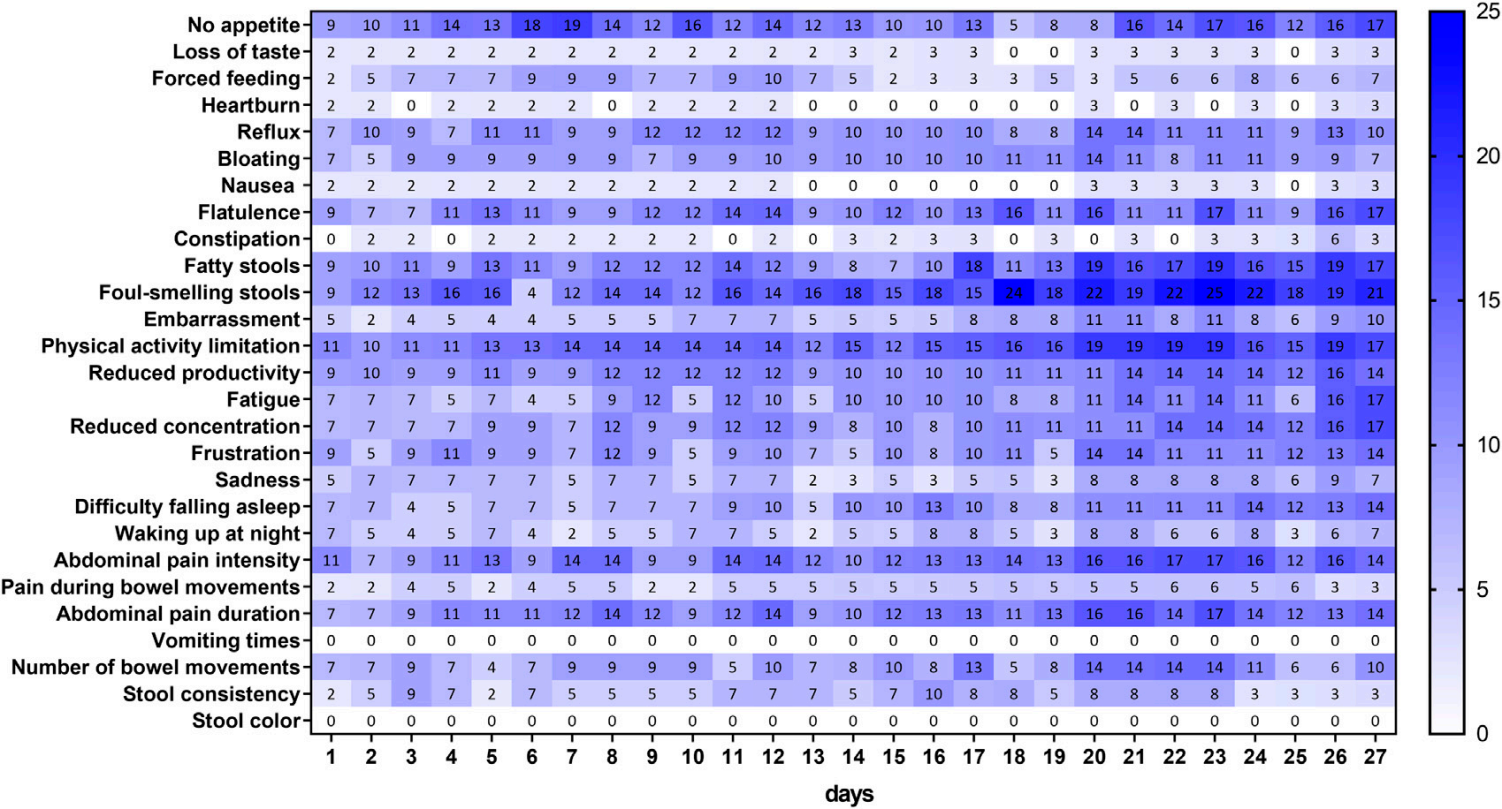
Proportion of pwCF reporting improvement for each item of the daily questionnaire with respect to their answers at baseline. Each percentage in the graph is relative to the total number of patients filling out the corresponding question on a specific day. Note that the questions regarding “Pain” and “Abdominal pain intensity” were condensed into a single conditional question.

Figure 2 shows the proportions of patients reporting improvement with respect to the corresponding baseline value for each of the 28 items included in the CFAbd-day2day^©^ over a maximum period of 4 weeks. As the question regarding “Pain intensity” depends on a positive answer for the occurrence of “Pain,” these two questions were condensed into a single conditional question referred to as “Abdominal pain intensity” throughout this article.

**FIGURE 3:**
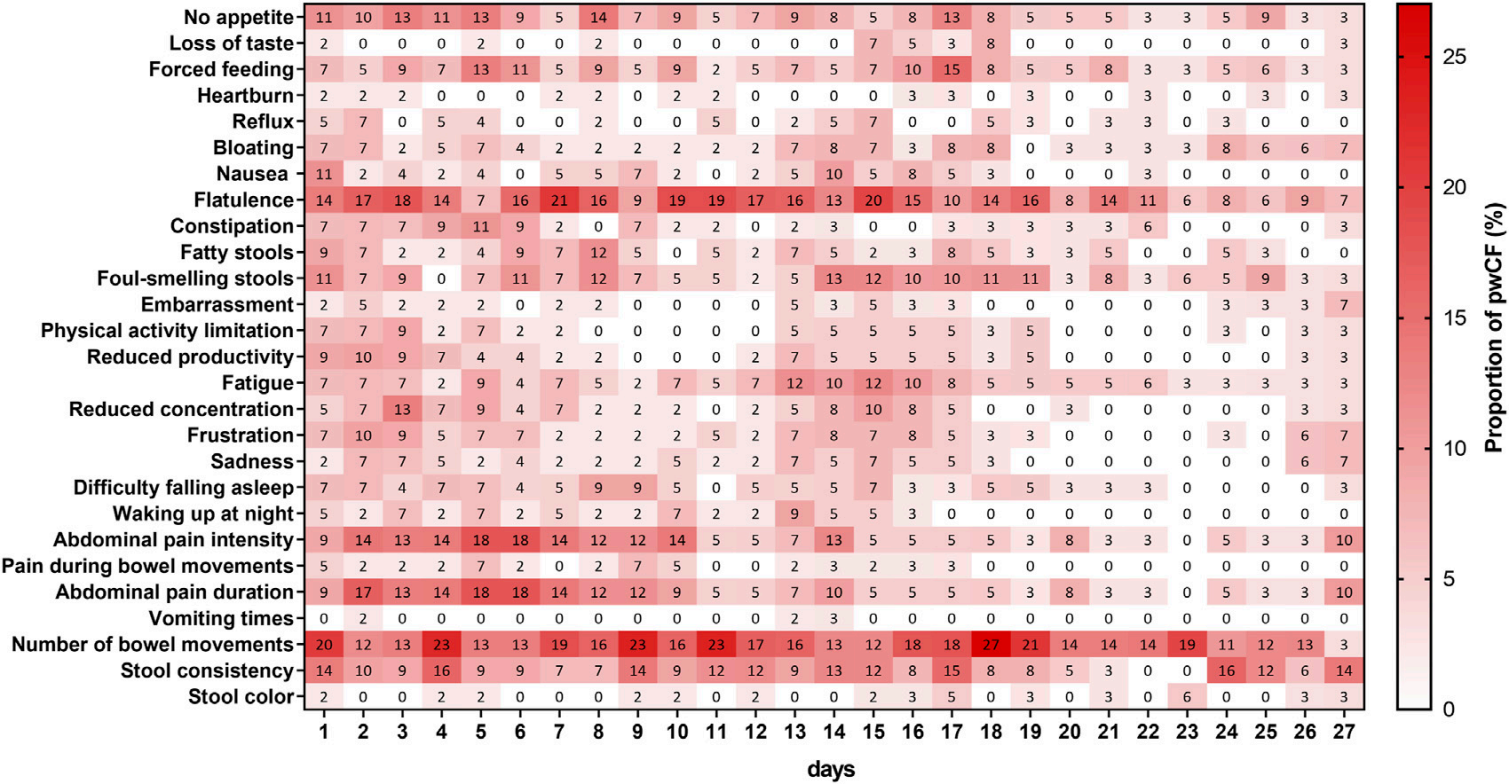
Proportion of pwCF reporting a worsening for each item of the daily questionnaire with respect to their answers at baseline. Each percentage in the graph is relative to the total number of patients filling out the corresponding question on a specific day. Note that the questions regarding “Pain” and “Abdominal pain intensity” were condensed into a single conditional question.

In two items, namely, “Vomiting times” and “Stool color,” no improvement with respect to baseline values was reported by the patients within this time frame. The highest proportion of patients (25%) reporting improvement was observed for the item “Foul-smelling stools” on day 23 after ETI therapy initiation. Other items for which the proportion of patients reporting improvement reaching almost the 20% level over this time frame were “No appetite” (19%), “Physical activity limitation” (19%), and “Fatty stools” (19%), with the former observed already on day 7 and the latter two from day 20 onward. On the other hand, symptoms for which the proportion of patients did not surpass the 10% level were “Loss of taste,” “Forced feeding,” “Heartburn,” “Nausea,” “Constipation,” “Sadness,” “Waking up at night,” and “Pain during bowel movements.”

The proportions of patients reporting worsening in symptoms within the first 4 weeks after ETI treatment initiation (Figure 2) were the highest for “Number of bowel movements,” for which 27% of patients on day 18 reported having a higher burden with respect to the time frame prior to ETI therapy. The second highest proportion was observed for “Flatulence,” reaching the maximum (21%) on day 7. Interestingly, after ETI therapy initiation, one patient reported an increase in the number of “Vomiting times,” and two patients reported worsening in their “Stool color.” Other items with relatively low worsening rates, as reported by the patients over this time frame, were “Difficulty falling asleep,” “Waking up at night,” “Physical activity limitation” (in the three items max: 9%), “Loss of taste,” “Bloating” (in both max: 8%), “Reflux,” “Embarrassment,” “Sadness,” “Pain during bowel movements” (in all max: 7%), and “Heartburn” (max: 3%).

Table 3 shows the averaged absolute daily changes in responses from patients reporting improvement or worsening in their GI symptoms (see Figures 2, 3) over the three considered periods of time: prior to ETI therapy and 1–14 and 15–27 days after ETI therapy initiation. According to this measure, variability levels for “Constipation” were significantly higher during the first 2 weeks after ETI therapy initiation. On the other hand, the variability in “No appetite,” “Reflux,” “Physical activity limitation,” “Abdominal pain intensity,” and “Abdominal pain duration” was significantly lower during the third and fourth weeks after ETI therapy initiation compared to the time frame prior to commencing ETI. However, compared to the 15–27-day time frame after ETI therapy initiation, the variability in “No appetite,” “Nausea,” “Reduced productivity,” “Fatigue,” “Reduced concentration,” “Difficulty falling asleep,” “Waking up at night,” “Abdominal pain intensity,” “Pain during bowel movements,” and “Abdominal pain duration” was significantly higher during the first 2 weeks of ETI treatment.

**TABLE 3:** Averaged absolute between-day variability in responses from patients reporting improvement or worsening in their GI symptoms after ETI initiation (see Figures 2, 3). The post-ETI therapy period was split into two 2-week time frames: 1–14 and 15–27 days after ETI therapy. Here, p1 indicates statistical significance for the comparisons between before and 1–14-day medians; p2 indicates statistical significance for the comparisons between before and 15–27-day medians; and p3 indicates statistical significance for the comparisons between 1–14-day and 15–27-day medians. No correction regarding multiple testing was conducted.

Averaged absolute between-day variability							
Item name	n	T_0_: 14 days before ETI [median, IQR]	T_1_: days 0–14 during ETI [median, IQR]	T_2_: days 15–28 during ETI [median, IQR]	p_1_ (T_0_–T_1_)	p_2_ (T_0_–T_2_)	p_3_ (T_1_–T_2_)
No appetite	17	0.2 [0–0.7]	0.4 [0.1–0.6]	0.1 [0–0.3]	0.82	**0.02**	**0.02**
Loss of taste	7	0 [0–0.2]	0 [0–0.1]	0.2 [0–0.2]	1	0.83	0.35
Forced feeding	12	0.3 [0–0.4]	0.4 [0.3–0.6]	0.3 [0–0.3]	0.4	0.33	0.07
Heartburn	7	0.3 [0.3–0.3]	0 [0–0.4]	0.3 [0.1–0.5]	1	1	0.58
Reflux	13	0.2 [0.2–0.5]	0.2 [0.1–0.3]	0 [0–0.3]	0.69	**0.02**	0.06
Bloating	14	0.2 [0–0.5]	0.2 [0–0.4]	0.1 [0–0.3]	0.31	0.72	0.84
Nausea	13	0 [0–0.2]	0.2 [0.1–0.3]	0 [0–0.1]	0.2	0.29	0.04
Flatulence	30	0.3 [0–0.7]	0.4 [0.1–0.6]	0.3 [0–0.5]	0.89	0.49	0.59
Constipation	13	0 [0–0.1]	0.2 [0.1–0.5]	0 [0–0.1]	**0.04**	0.59	0.23
Fatty stools	19	0.3 [0–0.6]	0.2 [0.1–0.7]	0.3 [0.2–0.6]	1	0.66	1
Foul-smelling stools	21	0.2 [0–0.6]	0.3 [0.1–0.6]	0.4 [0.1–0.5]	0.26	0.68	0.85
Embarrassment	10	0.4 [0.1–0.5]	0.1 [0–0.4]	0 [0–0.4]	0.53	0.14	0.37
Physical activity limitation	16	0.3 [0.1–0.6]	0.2 [0–0.3]	0 [0–0.2]	0.23	**0.03**	0.26
Reduced productivity	16	0 [0–0.2]	0.2 [0.1–0.3]	0 [0–0]	0.1	0.07	**0.01**
Fatigue	16	0.3 [0–0.5]	0.5 [0.2–0.8]	0.2 [0–0.4]	0.06	0.23	**0.01**
Reduced concentration	15	0.3 [0–0.4]	0.3 [0.2–0.5]	0.2 [0–0.3]	0.41	0.2	**0.03**
Frustration	12	0.2 [0–0.7]	0.2 [0.1–0.3]	0.1 [0–0.3]	0.73	0.08	0.11
Sadness	11	0.1 [0–0.4]	0.2 [0.1–0.4]	0.1 [0–0.2]	0.69	0.27	0.39
Difficulty falling asleep	14	0.1 [0–0.4]	0.2 [0.1–0.5]	0 [0–0.2]	0.36	0.14	**0.004**
Waking up at night	13	0 [0–0.3]	0.3 [0.3–0.5]	0 [0–0]	0.23	0.09	**0.01**
Abdominal pain intensity	23	0.5 [0.1–0.9]	0.4 [0.3–0.9]	0.1 [0–0.3]	0.59	**0.01**	**0.01**
Pain during bowel movements	7	0.1 [0–0.6]	0.2 [0.1–0.5]	0 [0–0]	0.69	0.1	**0.04**
Abdominal pain duration	23	0.4 [0.1–0.6]	0.4 [0.1–0.8]	0.1 [0–0.3]	0.81	**0.003**	**0.003**
Vomiting times	2	0 [0–0.1]	0.4 [0.4–0.5]	0 [0–0]	0.5	1	0.5
Number of bowel movements	34	0.5 [0.1–0.8]	0.5 [0.3–0.8]	0.3 [0.2–0.7]	0.85	0.32	0.09
Stool consistency	32	0.2 [0–0.7]	0.3 [0–0.8]	0.4 [0–0.7]	0.94	0.41	0.5
Stool color	9	0 [0–0.1]	0 [0–0.5]	0.2 [0–1]	0.28	0.14	0.37

*p*-values in bold represent statistically significant differences.

Comparing maximal absolute deviations from the median over the three time frames revealed that the maximum deviations in all items occurred within the first 2-week period. This was significant for items regarding “No appetite,” “Constipation,” “Fatigue,” “Waking up at night,” and “Abdominal pain duration” (Table 4). On the other hand, maxima in the item “Foul-smelling stools” observed within the period of first 2 weeks were significantly higher only when compared to the pre-ETI therapy time frame. The maxima for the five items, namely, “Nausea,” “Reduced productivity,” “Difficulty falling asleep,” “Abdominal pain intensity,” and “Pain during bowel movements,” within the first 2-week time frame were significantly higher compared to those observed within the third and fourth weeks after ETI initiation.

**TABLE 4:** Comparison of maximal absolute deviations from the median observed in each patient reporting the changes observed in Figures 2, 3 within the time frames prior to ETI therapy and after 14 and 28 days of ETI therapy initiation. The post-ETI therapy period was split into two 2-week time frames. Here, p_1_ indicates statistical significance for the comparisons between before and 14-day medians; p_2_ indicates statistical significance for the comparisons between before and 28-day medians; and p_3_ indicates statistical significance for the comparisons between 14-day and 28-day medians. No correction regarding multiple testing was conducted.

Maximal absolute deviations							
Item name	N	T_0_: 14 days before ETI [median, IQR]	T_1_: days 0–14 during ETI [median, IQR]	T_2_: days 15–28 during ETI [median, IQR]	p_1_ (T_0_ –T_1_)	p_2_ (T_0_–T_2_)	p_3_ (T1–T_2_)
No appetite	17	1 [1–2]	2 [1–3]	1 [0–1]	**0.03**	0.09	**0.001**
Loss of taste	7	0 [0–0.5]	0 [0–1]	1 [0.5–1.5]	1	0.3	0.3
Forced feeding	12	1 [0–2]	2 [1–3]	1 [0–2]	0.2	0.7	**0.05**
Heartburn	7	1 [0–2]	1 [0–1]	1 [1–1]	1	1	0.8
Reflux	13	1 [1–2]	1 [1–2]	0 [0–1]	0.8	0.07	0.1
Bloating	14	0.2 [0–1]	1 [1–2]	0.5 [0–2]	0.4	1	0.3
Nausea	13	1 [0–3]	2 [1–3]	0 [0–0]	0.3	0.2	**0.03**
Flatulence	30	1 [0–2]	1 [1–2]	1 [0–2]	0.6	0.7	0.4
Constipation	13	0 [0–1]	1 [1–2]	0 [0–1]	**0.02**	0.8	**0.02**
Fatty stools	19	1 [0–2]	1 [0.5–2]	1 [1–2]	0.3	0.1	0.7
Foul-smelling stools	21	1 [0–1]	2 [1–2]	1 [1–2]	**0.02**	0.2	0.6
Embarrassment	10	1 [0.1–2]	1 [0–2]	0.5 [0–2.5]	0.5	0.9	0.9
Physical activity limitation	16	1 [0.4–2]	1 [0–2]	0 [0–1]	0.6	0.2	0.08
Reduced productivity	16	1 [0–2]	2 [1–2]	0 [0–0.2]	0.2	0.5	**0.04**
Fatigue	16	1 [0.7–1.2]	2 [1–3]	1 [0–1]	**0.03**	0.5	**0.01**
Reduced concentration	15	1 [0-5–2.5]	1.5 [1–2]	1 [0–2.5]	0.9	0.5	0.3
Frustration	12	1 [0–2]	1 [1–2]	1 [0–1]	0.2	0.7	0.2
Sadness	11	1 [0–1.5]	1 [1–2.5]	0 [0–2.5]	0.2	0.8	0.2
Difficulty falling asleep	14	1 [0–1]	1.5 [1–2]	0.5 [0–1]	0.06	0.6	**0.02**
Waking up at night	13	0 [0–1]	2 [1–2]	0 [0–0]	**0.01**	0.3	**0.005**
Abdominal pain intensity	23	2 [0.5–2]	2 [1–3.5]	0 [0–1.5]	0.07	0.1	**0.007**
Pain during bowel movements	7	0.5 [0–2]	2 [1–2]	0 [0–0]	0.09	0.4	**0.03**
Abdominal pain duration	23	1 [0.5–2]	2 [1–3]	0 [0–2]	**0.02**	0.2	**0.004**
Vomiting times	2	1 [0.5–1.5]	3.5 [3–4]	0 [0–0]	1	1	0.5
Number of bowel movements	34	2 [1–2]	2 [1–2]	1 [0.2–2]	0.7	0.1	0.08
Stool consistency	32	1 [0–3]	1.5 [0–3]	1 [0–3]	0.3	0.6	0.3
Stool color	9	0 [0–0]	0 [0–3]	3 [0–3]	0.3	0.1	0.7

*p*-values in bold represent statistically significant differences.

### 3.2 Response to ETI therapy initiation

Effects of ETI therapy assessed with averaged CFAbd-day2day^©^ responses revealed a highly significant decrease in the median total score (*p* = 0.00001). Changes in four domains, namely, “Pain,” “GERD,” “Disorders of bowel movement,” and “Quality of life impairment,” were statistically significant (*p* = 0.0003, 0.01, 0.02, and 0.01, respectively), although medians for “GERD” and “Quality of life impairment” resulted equal for the two time frames (Table 5).

**TABLE 5:** Changes in averaged CFAbd-day2day^©^ responses after ETI therapy initiation, comparing the cumulative burden of symptoms during the 14-day period prior to (T_0_) and the second 14-day period after initiation of the new therapy (T_2_: days 15–28).

		T_0_: 14 days before ETI [median, IQR]	T_2_: days 15–28 during ETI [median, IQR]	*p*
Total cumulative CFAbd-day2day^©^ scores		8.9 [2.8–15.7]	4.7 [1.3–10]	**0.00001**
Five domains of the CFAbd-Score	Pain	6.7 [0–20]	0.0 [0–6.7]	**0.0003**
	GERD	0.0 [0–26.7]	0.0 [0–6.7]	**0.01**
	Disorders of bowel movement	10.0 [2.5–22.5]	7.5 [0–15]	**0.02**
	Disorders of appetite	4.0 [0–28]	0.0 [0–20]	0.1
	Quality of life impairment	0.0 [0–5]	0.0 [0–0]	**0.01**

*p*-values in bold represent statistically significant changes between cumulative scores before and after ETI therapy.

### 3.3 Correlation with CFAbd-Score^©^

**FIGURE 4:**
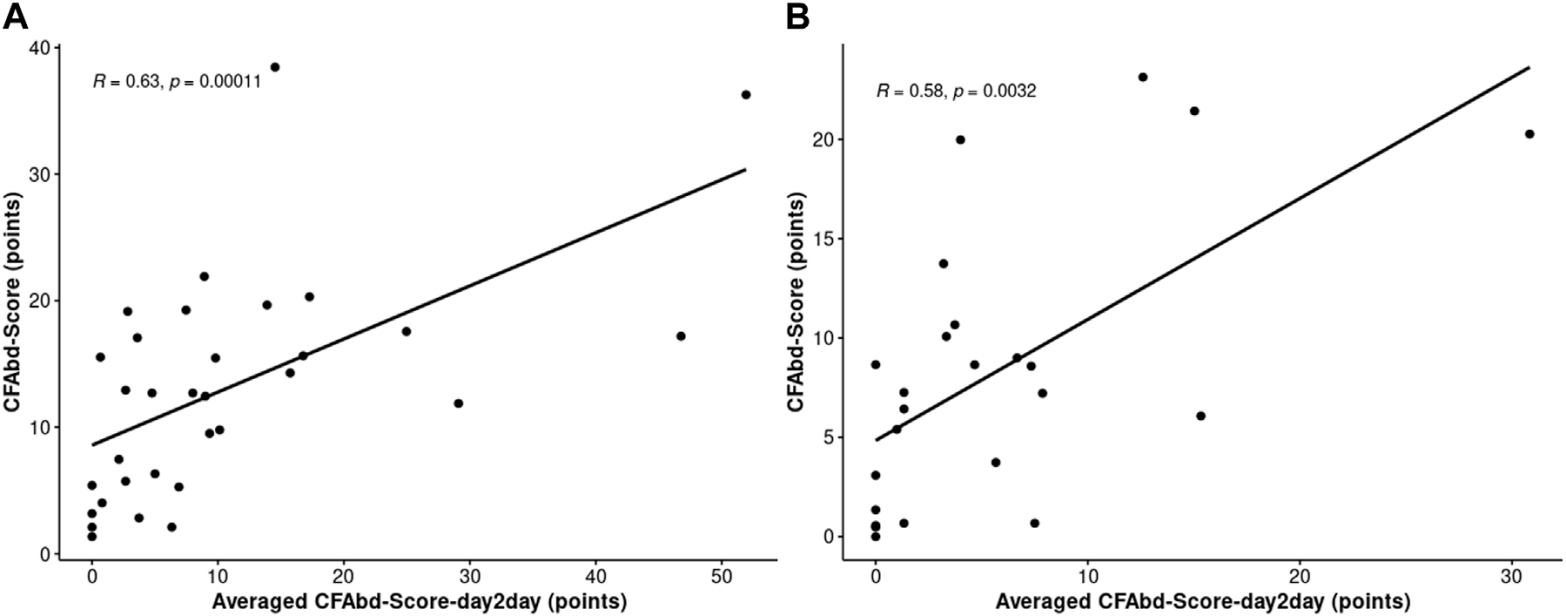
Coefficients between averaged CFAbd-day2day^©^ responses and retrospective CFAbd-Score^©^ covering 2 weeks prior to ETI **(A)** and 3–4 weeks after ETI initiation **(B)**. The number of patients who completed both CFAbd-day2day^©^ and retrospective CFAbd-Score^©^ covering 2 weeks prior to ETI was n = 32. The number of patients who completed both CFAbd-day2day^©^ and retrospective CFAbd-Score^©^ covering 3–4 weeks after ETI initiation was n = 24.

Averaged CFAbd-day2day^©^ responses of pwCF and retrospective CFAbd-Score^©^ covering the 2 weeks prior to ETI initiation showed a strong correlation (Pearson r = 0.63; n = 32; *p* < 0.001) (Figure 4A). On the other hand, the correlation between both domains (Pearson r = 0.58; n = 24; *p* < 0.01) was slightly lower at 3–4 weeks (Figure 4B).

## 4 Discussion

With availability of highly effective CFTR modulators like ETI, a game changer in treatment of CF, it is essential to thoroughly identify the spectrum of effects of the novel medication (Bell et al., 2019; King et al., 2021). At the same time, it may be a historic opportunity to record the burden of symptoms in pwCF who, at baseline, are still naïve for game-changing medications like HEMTs. Therefore, in the present study, we prospectively assessed AS changes on a daily basis before and immediately after the initiation of a new, highly effective CFTR-modulating ETI therapy in 45 pwCF using a novel PROM, the novel CF-specific GI-symptom diary “CFAbd-day2day^©^”. The development of the new PROM was based on the CFAbd-Score, the first CF-specific GI PROM designed and validated following FDA guidelines (Tabori et al., 2017a; U.S. Department of Health and Human Services Food and Drug Adminitration, 2019; Jaudszus et al., 2019). To the best of our knowledge, this is the first publication including a diary that closely records CF-specific AS, developed following FDA guidelines and COSMIN methodology for the development of a PROM (Mokkink et al., 2010; U.S. Department of Health and Human Services Food and Drug Adminitration, 2019).

Altogether, the results obtained using the CFAbd-day2day^©^ cumulatively calculated for each fortnight, i.e., the fortnight before ETI initiation and the second fortnight after ETI initiation, were in accordance with our previously published results obtained using the CFAbd-Score in 107 pwCF from Germany and the UK: GI symptoms significantly decreased after 2–4 weeks, which was consistent with the previous findings using the 28-item CFAbd-Score prior to and 4 and 26 weeks after initiation of ETI, retrospectively capturing the burden of GI symptoms during the preceding 14 days (Mainz et al., 2023; Mainz et al., 2022). This is also evident in the resulting high correlation between the averaged CFAbd-day2day^©^ and the retrospective CFAbd-Score^©^ from a subgroup of pwCF who had concomitantly completed both questionnaires (Figure 4). Our new findings with the CFAbd-day2day^©^ reveal that the initiation of ETI is followed by changes in abdominal symptomatology according to quite individualized profiles, substantially differing from one pwCF to another receiving the new therapy. The extent of short-term changes in GI symptomatology is exemplified by the individual CFAbd-day2day^©^ record from a single CF patient (Figure 1). In other patients included in this study, however, some of these complaints occurred less frequently during these time frames.

Despite the highly individual pattern of changes, trends in the overall cohort are visible in Figures 2, 3, revealing that some symptoms appear, increase or decline more frequently in a proportion of patients over time. For instance, abdominal pain, including its duration and intensity, increased in up to 18% of patients during the first 10 days of ETI initiation, together with an increase in flatulence in 21% of patients about day 7. Then, after 11–15 days of ETI initiation, the proportion of patients reporting pain symptoms markedly decreased to 0%–10%. Furthermore, flatulence appeared to improve during the last observational week (days 15–27) in many patients, together with a higher number of patients reporting a decrease in fatty and foul-smelling stool.

“Between-day variability” and “maximal absolute deviations from the median” in CFAbd-day2day^©^ items reveal some statistically significant changes in the patterns of variability. Again, the crucial symptoms of abdominal pain, including its intensity and duration, as well as pain during bowel movements, reveal a significant reduction in the variability over the late observational period.

Notably, the present study included a relatively high proportion of younger pwCF, due to the approval of ETI in pwCF between 6 and 12 years of age carrying a F508del mutation during the study period (median age: 10 years, range: 6–55 years). According to our previous studies with the CFAbd-Score, children report significantly more often on abdominal pain, whereas adults complain significantly more often about gastroesophageal reflux (Tabori et al., 2017a; Jaudszus et al., 2019). Accordingly, including a higher percentage of adult pwCF or even patients with more advanced disease progression results in a different pattern of symptoms, specifically in regard to GERD and vomiting, which were rare symptoms in our cohort (Tabori et al., 2017a; Mainz et al., 2018; Jaudszus et al., 2019).

Calendars documenting specific patterns of symptoms are used as a golden standard in the care of patients suffering from complaints such as recurrent headaches or chronic abdominal pain, including Crohn’s disease, ulcerative colitis, or irritable bowel disease. However, the pattern of AS in pwCF has been found to be rather specific due to patterns in CFTR deficiencies in the exocrine and endocrine pancreas, small and large intestines, and bile ducts (Ooi and Durie, 2016). Accordingly, PROMs developed for other non-CF-specific abdominal pathologies, like irritable bowel disease, chronic inflammatory bowel diseases, non-CF-related GER, pancreatitis, or constipation, may not be adequately sensitive to the CF-specific pattern of GI symptoms (Hayee et al., 2019). In our eyes, these limitations are reflected, for instance, in the lack of sensitivity to detect ETI effects in large multicenter trials using the PAGI-SYM, PAC-SYM, or PAC-QoL (Schwarzenberg et al., 2022). Although significant improvements in symptoms were identified therein, such changes were estimated to be too small to achieve clinical relevance, according to the authors. In contrast, CF-specific AS assessed using the CFAbd-Score declined in 107 pwCF from Germany and the UK from a mean of 14.9 to 10.6 pts during 24 weeks of ETI (*p* < 0.05), similar to the five domains of Pain, GERD, Disorders of bowel movement, Disorders of appetite, and GI-related QoL (Mainz et al., 2022). Likewise, a similar improvement was observed in 108 pwCF from Ireland and the UK, assessed using the CFAbd-Score during a new therapy with ETI (Mainz et al., 2023). Therefore, the level of changes captured with the CF-specific CFAbd-Score can be considered clinically relevant (Caley et al., 2023b).

To the best of our knowledge, the study presented here investigates for the first time AS recorded in detail after ETI initiation using a CF-specific validated diary approach. Before HEMT approval, previous studies assessing GI symptoms in pwCF focused on either abdominal pain with non-CF-specific PROMs or did not report information regarding the methodology or specific content and design of implemented diaries (Elliott et al., 1992; Obideen et al., 2006; Munck et al., 2012; Van Biervliet et al., 2018). For instance, Elliott et al. (1992) compared the effects of different PERTs on GI complications using some type of a symptom diary. They reported pwCF to prefer microcapsules as PERT because these appeared to cause lower rates of abdominal symptoms and the dosage included fewer capsules. However, no differentiation of abdominal symptoms or further information about the employed diary was provided (Elliott et al., 1992). Two other studies analyzed abdominal pain in pwCF using a pain diary. One investigated recurrent abdominal pain in n = 8 pediatric pwCF, and the other observed the effect of nocturnal hydration on abdominal pain in n = 9 pwCF (Obideen et al., 2006; Munck et al., 2012). In the latter, the pain diary assessed the frequency, medications, and pain intensity on a visual analog scale, whereas the former used different PROMs for pain measurement (Eland Pain location, pain intensity measured by Faces Pain Scale—Revised (FPS-R), McGill Emotional Status, R-CMAS anxiety score, and health-related quality of life (CF-QOL)) at the first study visit (Obideen et al., 2006; Munck et al., 2012). In both studies, however, further information regarding the content of the diary or information about a possible validation was missing. Recently, Van Biervliet et al. (2018) used a diary to explore the effects of probiotics on GI symptoms and on intestinal flora in n = 31 pwCF. Outcomes included fecal calprotectin, pulmonary function, nutritional status, and AS assessed with a diary, which queried abdominal pain, stool frequency, and treatment changes. Again, further information about the diary was not provided.

Diaries are favourable when short term changes are expected. This applies to our setting assessing changes in AS during ETI initiation, which was motivated beforehand by numerous pwCF’s reports on short-term changes in GI symptoms, often commencing hours after receiving the first dosage of HEMT. In the development process of the novel PROM, the 28 items included in the CFAbd-day2day^©^ were identified as highly relevant by pwCF, proxies, and CF caregivers of different professions (community voice), who were repeatedly consulted. Consequently, the questionnaire was observed to have high acceptance rate.

The results presented in this publication fulfil essential steps in the validation process of the CFAbd-day2day^©^. They reveal that the PROM is sensitive to detect symptom changes, e.g. those caused by a newly initiated therapy. At present, further steps to validate the CFAbd-day2day^©^ are in progress, including analyses of convergent validity with the CFAbd-Score and AS dynamics in pwCF suffering from CF-related conditions like constipation, DIOS, GERD, CF-related diabetes (CFRD), and liver disease.

In summary, implementation of the novel CFAbd-day2day^©^ during a new therapy with the HEMT ETI provides new real-world insights relevant for the CF community and healthcare providers. Furthermore, the symptom diary can be implemented in routine care of pwCF suffering from GI symptoms in order to follow up their dynamics in daily life, as well as the effects of therapeutic interventions. Accordingly, the CF-specific CFAbd-day2day^©^ may facilitate the identification of critical GI complications that may require the individual with CF to consult the attending CF center. Analogous to the CFAbd-Score, the PROM is being translated to other languages and will be implemented in international studies.

## Data Availability

The raw data supporting the conclusion of this article will be made available by the authors, without undue reservation.
